# Perspectives on Lower Extremity Peripheral Artery Disease: A Qualitative Study of Early Diagnosis and Treatment and the Impact of Health Disparities

**DOI:** 10.1089/pop.2023.0095

**Published:** 2023-11-24

**Authors:** Cori Grant, John K. Cuddeback, Olamide Alabi, Caitlin W. Hicks, Kay Sadik, Elizabeth L. Ciemins

**Affiliations:** ^1^ AMGA (American Medical Group Association), Alexandria, Virginia, USA.; ^2^ Division of Vascular Surgery and Endovascular Therapy, Emory University School of Medicine, Atlanta, Georgia, USA.; ^3^ Division of Vascular Surgery and Endovascular Therapy, Johns Hopkins University School of Medicine, Baltimore, Maryland, USA.; ^4^ Janssen Scientific Affairs, LLC, Titusville, New Jersey, USA.

**Keywords:** peripheral artery disease, primary care, vascular medicine, screening, treatment

## Abstract

Lower-extremity peripheral artery disease (PAD), the accumulation of atherosclerotic plaque in the arteries of the legs, causes substantial morbidity and mortality. Frequent under- and delayed diagnosis result in poor outcomes, disproportionately affecting individuals from racial and ethnic minority groups. To understand barriers to early detection and treatment and factors contributing to disparities, American Medical Group Association (AMGA) conducted roundtable discussions and semistructured interviews in 2021. Eighteen participants discussed PAD evaluation, diagnosis, early medical management, and disparities in care. A qualitative case study approach and data reduction methods were used to generate themes, draw conclusions, and make actionable recommendations. Identified themes included lack of (1) prioritization of PAD for population health; (2) engagement of primary care providers in early evaluation and referral; (3) “ownership” of lower-extremity PAD within health systems; and (4) focus on disparities in care. Participant solutions included (1) financial impact of early PAD management, in the context of value-based payment; (2) embedding an advanced practice provider into a vascular surgery practice to facilitate evaluation and provide medical therapy; and (3) leveraging care coordination, multidisciplinary clinics, and telehealth technology to provide comprehensive care for patients with PAD and address disparities. A deliberate focused effort is necessary to close gaps and the accompanying disparities in early evaluation, diagnosis, and treatment for people with lower-extremity PAD. The authors describe 3 models that can be emulated to improve care for this high-risk population. With improved reimbursement and better medical therapies, now is the time to focus on early diagnosis and management of PAD.

## Introduction

**FIG. 1.**
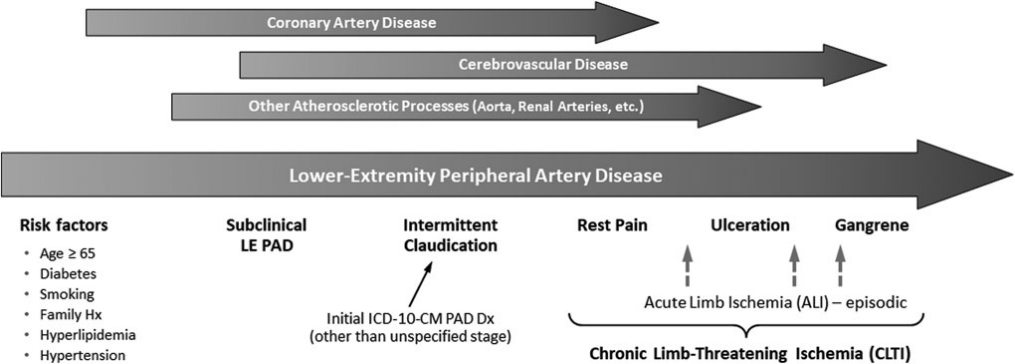
Underlying pathophysiology and manifestations of PAD. Although the atherosclerotic disease process that underlies PAD is generally progressive and is often present in multiple vascular beds, the symptoms and clinical presentation of lower-extremity PAD are notably variable and individual patients may not experience the classical sequence of symptoms. Observational studies using EHR data are limited to using ICD-10-CM diagnosis codes, which are defined based on a strict progression of manifestations. CLTI, generally associated with the appearance of rest pain, indicates more advanced pathophysiology. Multiple episodes of ALI may occur at any time during the phase of CLTI. [The term CLTI was adopted in 2019.^2^ Before that, the term critical limb ischemia (CLI) was commonly used.]. ALI, acute limb ischemia; CLI, critical limb ischemia; CLTI, chronic limb-threatening ischemia; EHR, electronic health record; ICD-10-CM, International Classification of Diseases, Tenth Revision, Clinical Modification; PAD, peripheral artery disease.

Peripheral artery disease (PAD) is a chronic atherosclerotic disease of the lower-extremity arteries resulting in reduced blood flow. The manifestations of PAD and its complications include intermittent claudication, ischemic rest pain, ulceration, and gangrene. Early PAD includes asymptomatic disease and intermittent claudication—before ischemic rest pain, which is classically regarded as the earliest indication of chronic limb-threatening ischemia.^1^ (Fig. 1 depicts the generally progressive underlying disease process.)

In 2021, the American Heart Association (AHA) reported 6.5 million documented cases of lower-extremity PAD among US adults ≥40 years old.^2^ However, many patients do not present with classic symptoms and, therefore, the actual prevalence of PAD may be closer to 12 million.^3^ This variation in presentation can lead to delays in diagnosis.^4^ Earlier evaluation and treatment are crucial to reduce the high rates of potentially preventable morbidity, amputation, and death.^5^

The comprehensive management of early PAD, including exercise, lifestyle changes, and medication, is well supported by evidence and is consistent across multiple professional guidelines [AHA,^6,7^ Society for Vascular Surgery (SVS),^8^ and the Canadian Cardiovascular Society (CCS)^9^]. The use of invasive procedures for revascularization, however, should be based on careful clinical consideration of an individual's disease state.^10–12^ Overall, the care of a patient with PAD is complex; it requires a nuanced balance of screening, diagnosis, and treatment to optimize patient outcomes.

PAD disproportionately affects people from racial and ethnic minority groups and those who are socioeconomically disadvantaged. Disparities (eg, differences in patient awareness of PAD) are a significant contributor to delayed diagnosis.^13^ Prevalence of risk factors for PAD, including diabetes and smoking, is higher among African Americans in the United States. One in 3 Black individuals will develop PAD in their lifetime, compared with 1 in 5 White individuals.^14^

Mortality among patients who undergo amputation resulting from PAD may be as high as 50% at 1 year.^15^ Black race is an independent predictor for lower extremity amputation among individuals with PAD.^16^ Black individuals have amputation rates up to 4 times greater than non-Hispanic White individuals.^17^ These factors suggest that this patient population may be diagnosed later or receive less effective treatment.

There are 3 timely reasons to pay attention to early identification of lower-extremity PAD and to make it a population health priority. First, Medicare added coverage in 2017 for supervised exercise therapy, in addition to smoking cessation counseling, for those diagnosed with lower-extremity PAD.^18,19^ Another reason to intensify PAD early detection efforts is the recent addition of newly approved medical therapy for patients with PAD.^20,21^ Third, with many health systems focusing on improving health equity, lower-extremity PAD presents an opportunity for substantial progress.

Limited awareness of PAD among patients and clinicians and inequitable delays in diagnosis and treatment make this an important area of study.^4,14,16,17^ To understand why PAD is underdiagnosed and the practices that may result in inequitable care, the team spoke with primary care providers (PCPs) and cardiovascular specialists across the United States. The goal was to understand current clinical practice in the evaluation and treatment of lower-extremity PAD, including barriers and opportunities to improve care, with a focus on existing disparities and potential solutions.

## Methods

### Study sample

Eighteen participants were recruited from organizational members of American Medical Group Association (AMGA), a nonprofit trade association whose mission is to support its members through advocacy, education, quality improvement, and research. AMGA's primary focus is to help its members move from volume to value by focusing on population health and the quintuple aim: better health, better care, lower cost, provider and staff wellness, and health equity.^22^

Thirteen geographically diverse organizations in the United States participated (see Table 1). Participants included representatives from primary care, vascular medicine, vascular surgery, cardiology, podiatry, pharmacy, clinical operations, and population health. The WCG Institutional Review Board determined this study exempt from human subjects' protections review.

**Table 1.** Participant Characteristics (*N* = 18)

	Male (*n* = 11)	Female (*n* = 7)	Northeast (*n* = 4)	Southeast (*n* = 6)	Southwest (*n* = 1)	Midwest (*n* = 5)	West (*n* = 2)
Primary care physician	2	2		2	1	1	
Cardiology^a^	2	1		1			2
Vascular surgery	3	2	2	2^b^		1	
Vascular medicine	3		1			2	
Other^c^	1	2	1	1^d^		1	

^a^ Includes interventional cardiology.

^b^ Includes 1 clinician in private practice.

^c^ Clinical pharmacist, director of managed care, podiatrist.

^d^ Organization is a large multispecialty practice.

### Quantitative insights

To stimulate discussion in the roundtables and interviews, AMGA conducted an analysis using data from the Optum Labs Data Warehouse, a longitudinal real-world data asset with deidentified administrative claims and electronic health record data, to explore the timeliness of diagnosing PAD. This analysis showed that 64% of patients with a diagnosis of lower-extremity PAD with ulceration had not had any prior PAD diagnosis (AMGA Analytics internal analysis, unpublished, 2022) (see Table 2).

**Table 2.** Evidence of “Late” Diagnosis of Lower-Extremity Peripheral Artery Disease

“Stage” of patient's first ICD-10-CM diagnosis code for lower-extremity PAD	Proportion of patients (%)	Median time from first PAD diagnosis until diagnosis of PAD with ulceration (days)
PAD with ulceration	64	—
PAD with rest pain	5	103
PAD with intermittent claudication	17	195
PAD, unspecified stage	14	—
Total	100	—

Data were sourced from the Optum Labs Data Warehouse, a longitudinal, real-world data asset with deidentified administrative claims and EHR data. Among 31,572 patients who ultimately had a diagnosis of lower-extremity PAD with ulceration. Data from the Optum Labs Data Warehouse for 31,572 patients who had an ICD-10-CM diagnosis code for lower-extremity PAD with ulceration (on their EHR problem list or on a claim for a clinical encounter) illustrates the delay in diagnosis of PAD. This table displays the proportion whose *first* diagnosis code for lower-extremity PAD was associated with each “stage” of the disease. Sixty-four percent had no diagnosis of lower-extremity PAD before the diagnosis associated with ulceration. For those whose initial PAD diagnosis was associated with an earlier stage (rest pain or intermittent claudication), the median time from that initial diagnosis to the diagnosis indicating ulceration was strikingly short.

EHR, electronic health record; ICD-10-CM, International Classification of Diseases, Tenth Revision, Clinical Modification; PAD, peripheral artery disease.

### Qualitative research

Two 60-minute virtual roundtable discussions were conducted by AMGA researchers in September and October 2021 followed by six 60-minute semistructured interviews in November 2021. Roundtable discussion and interview guides were developed and shared with participants in advance. Topics included lower-extremity PAD evaluation and diagnosis, medical management, and disparities in diagnosis and treatment. Discussions and interviews were recorded and transcribed.

### Analysis

The analysis used a qualitative case study approach^23^ and data reduction methods to generate themes and draw conclusions. The case study methodology is appropriate to explore a phenomenon within a specific context through various lenses or perspectives. Two qualitative researchers participated in all discussions and interviews, reviewed all transcripts, and independently generated initial themes from the data. Through an iterative process, themes were compared and discussed until consensus was reached.

Additional data validation was conducted by 1 researcher by checking defined themes using the raw transcripts loaded into ATLAS.ti version 9 for Windows (Atlas.ti Scientific Software Development GmbH, Berlin, Germany). Finally, themes and conclusions were shared with all participants, who verified the results (member-checking). No alterations were necessary following this process.

## Results

Discussions about early detection and diagnosis, adequate management, and inequitable care and outcomes led to the identification of 4 primary themes: (1) PAD is not a population health priority; (2) PAD is not a major focus for PCPs; (3) “ownership” of lower-extremity PAD is lacking within integrated health systems; and (4) disparities in care are exacerbated by lack of prioritization and ownership by health systems, PCPs, and specialists. Each is described in detail hereunder, followed by 3 case studies that provide viable solutions to these challenges. (Table 3 provides additional supporting quotes and information drawn from the data for the identified themes.)

**Table 3.** Summary of Themes, Related Dimensions, and Select Participant Quotes and Supporting Information

Theme	Dimension	Participant quotes and supporting information
PAD is not a population health priority for organizations	Low reimbursement	• Insurers do not reimburse for screening among those who are asymptomatic. • Supervised exercise and smoking cessation have not been well reimbursed until recently.
	Lack of accountability/education	• “[Education] should include emergency room doctors…when patients come to the hospital with leg pain, they often get evaluated for a DVT and nothing on the arterial side. Upon evaluation … I discover they have arterial disease and not … DVT.”—cardiologist • Lack of specific education on early PAD during training for clinicians and APPs. • Young smokers are being missed with their first diagnosis of atherosclerotic disease as PAD.
	Difficult to diagnose	• Physical examination and history taking are critical, but they are often overlooked at all levels of care. • Patients often limit their physical activity to avoid pain making it difficult to obtain a good history. • Structure of diagnosis codes confounds symptoms (intermittent claudication, rest pain) with diagnosis, limiting the ability to study, understand, and monitor lower extremity PAD.
Detection of PAD is not a focus for primary care	Lack of time and space for screening tests	• Staffing and space limitations in primary care make ABIs difficult to administer. • Primary care physicians may screen new patients, but they need reminders for annual evaluation. • Reminders focus on established quality measures and seldom include PAD, which does not have quality measures.
	Lack of guidelines/little benefit/limited education	• No reason to screen for PAD because there has been no evidence-based medical therapy for PAD until recently. • PCPs interpret the USPSTF “I” statement as discouraging screening of asymptomatic patients. • “There is…lack of awareness … there needs to be more … responsibility … to not only diagnose the disease but to pursue adequate avenues for those patients to be treated effectively.”—vascular medicine physician
Lack of ownership for PAD care		• Cardiologists focus on coronary arteries; lower extremities don't get a lot of attention. • Endovascular treatments are owned by different specialties in different organizations. • Cardiology and vascular surgery don't have time to focus on prevention, for example, smoking cessation, exercise.
Disparities in care	Lack of access to high-quality care	• “Once I do diagnose claudication, I order the ABI. At that point the health care disparities kick in for the folks that … don't have the … resources to get to that additional appointment.”—primary care physician • “I hand pick cardiologists because there are many barriers, including cultural aspects of specialty clinics that make my patients of color or who are low literate or low resource not even want to go there because they don't feel heard.”—primary care physician • “Anecdotally, … when I think about the people … who are inappropriately receiving a large volume of procedures … the non-ambulatory patient that probably should not have had a procedure in the first place. I cannot think of anyone who was not Black or Brown in that situation. These are often people who are at community sites, usually office based labs and they are getting procedure after procedure after procedure.”—vascular surgeon
	Social determinants of health	• “[patients] have difficulty with transportation or taking time off work. By consolidating visits … you get over a lot of those barriers.”—vascular surgeon • Transportation provided by insurer can take 7 working days to schedule if there are delays.
	Lack of patient education	• Patient education may help mitigate disparities because educated patients will ask questions. • Patients think leg pain is a nerve or muscle because they are not aware of PAD.

ABI, ankle-brachial index; USPSTF, United States Preventive Services Task Force.

### PAD is not a population health priority for health systems

Participants reported that under fee-for-service payment, which still dominates in much of the United States, providers are unable to share in the long-term savings that will result from reducing the risk of disease progression through early identification and management of PAD. Although there is still benefit to the patient, there is no financial incentive for the provider organization to take a population health approach. Although treatment of PAD is now often reimbursed, payment rates are modest for the time-intensive vascular medicine services required for evaluation (ankle-brachial index [ABI]), as well as for supervised exercise therapy and smoking cessation counseling.

Participants also noted the lack of quality measures for PAD as a reason for lack of prioritization. Organizations are instead prioritizing other conditions for which they are being held accountable, such as diabetes and hypertension. Participants added that in addition to being a component of provider compensation, such measures also help providers and organizations identify areas for improvement as well as disparities in care. Without recognized clinical performance measures, there may be little incentive to monitor the quality of care for PAD including evaluation, diagnosis, and treatment. One participant noted:

There is no PAD [quality] measure, so it is not an institutional priority outside of billing.

Even if adequately incentivized, clinicians reported that diagnosing lower-extremity PAD is challenging. Presentation of this condition is highly variable, as patients have often adjusted their lifestyle to avoid pain, making it difficult to elicit a reliable history.

For these reasons, in addition to the strain and burden providers have felt during the COVID-19 pandemic, health systems are left with little incentive to prioritize early detection of PAD or to develop protocols and processes to assist clinicians in its management.

### PAD is not a major focus for PCPs

All participants, including PCPs, acknowledged that early detection and diagnosis of PAD is not a major focus for PCPs. They said providers have traditionally seen little benefit in diagnosing lower-extremity PAD when they are already treating atherosclerosis in other vascular beds. This additional diagnosis would not have changed the course of therapy. One PCP said,

… until two years ago … if you had hypertension … [or] diabetes … you were on a statin, and so there wasn't really that compelling reason to … diagnose … PAD … [because it wouldn't] alter their quality or quantity of life.

However, it was also noted that given new treatments and additional reimbursement, having a diagnosis focuses attention on interventions that may help prevent or delay serious complications.

Another reported issue in primary care was the lack of time for tasks like careful foot examinations and assessment of pedal pulses, which are often key to early detection of PAD. Exacerbating these barriers, a lack of internal follow-up processes was cited as a reason for missed annual evaluations for at-risk patients.

Participants reported that “ambiguous” clinical guidelines may contribute to a lack of action by PCPs. The United States Preventive Services Task Force (USPSTF) maintains that there is “insufficient evidence” to conduct ABI tests among asymptomatic individuals.^24^ Clinical leaders said this is often interpreted as a recommendation *not* to test, leading to underdiagnosis:

USPSTF [statement] is a major limitation … it's important to differentiate screening (for a patient who does not have signs/symptoms) from confirmation diagnosis in patients with a sign/symptom and risk factors for PAD.

Although European or other international clinical guidelines may state otherwise, according to the participants, US clinicians place greater stock in guidelines originating in the United States.

Although it is technically feasible to perform an ABI in a primary care setting, it is not practical in most offices, pressed as they are for space, time, and skilled staff. An ABI generally requires referral to a vascular laboratory. One vascular surgeon mentioned the lasting impact of the COVID-19 pandemic and said,

I'm always reluctant to say that primary care should “own” another disease … they are already way overloaded.

One participant's health system, which is the subject of Case Study 1, is using a newer device to make screening in primary care more practical and has created management guidelines to identify PAD patients.

### “Ownership” of lower-extremity PAD is lacking within integrated health systems

A practical challenge identified by participants is that there is no broadly established medical specialty that is focused on identification of PAD. In most health systems, early PAD lacks a home and thus a champion for timely diagnosis and medical therapy to prevent the progression of disease. PAD falls within cardiovascular medicine, but participants noted that most cardiologists are focused on the coronary arteries, and there is no procedure for early PAD. There are fellowships in vascular medicine, but it is not a distinct subspecialty of internal medicine. As a cardiologist/vascular medicine specialist said,

… people's feet … [are] frequently overlooked … whether they be in primary care or in … cardiology. [Cardiologists] tend to think about the heart and forget about all the rest of the blood vessels.

Patients are often referred to vascular surgery for evaluation, but those with early disease need medical management, not endovascular or surgical intervention.

Vascular surgery owns the advanced form of PAD, they're interested when it's time to put in a stent or do a bypass surgery, which is a small fraction of PAD and is the part we really want to try to avoid.

Participants noted the additional complication in some organizations that endovascular procedures for later-stage PAD may be “owned” by multiple specialties—cardiology, interventional radiology, or vascular surgery.

Even when a procedure is performed, providers reported that follow-up is often lacking, related to a lack of ownership of holistic care for the patient, beyond the procedure itself. In an example from an interventional cardiologist, a patient underwent an amputation on one leg, and no attention was paid to the other leg.

… we still have far too many amputations … about half of those patients … have no vascular evaluation whatsoever, prior to the amputation or afterwards … that should be at the very least a mandate—for vascular evaluation of any patient who has an amputation, be [it] before or after, to at least try to prevent … the second amputation, which we know is very likely to follow.

Several participants mentioned podiatry as another potential champion (and mechanism) for early detection of PAD. But the podiatrist participant said his discipline is often focused on ankle surgeries and thus not available for management of patients with PAD.

Participants agreed that these collective challenges often result in patients being referred too late for evaluation. When patients are referred, it is often to vascular surgery, where the priority is evaluation for a potential procedure, not diagnosis and medical management of early disease. One vascular specialist stated,

I [can] think of … five people over the last … three or four years, who didn't see … [a] primary care physician, wound care clinic … [or] a podiatrist … while they had [a] wound or … discoloration that eventually became gangrene before they actually got to us.

### Racial disparities in care are exacerbated by lack of prioritization and ownership by health systems, PCPs, and specialists

The final theme that emerged was how delays in diagnosis and management result in disparities in care. These disparities are exacerbated by all the issues described earlier. Although there are challenges to referring patients in general, PCPs reported being particularly reluctant to refer patients from ethnoracial minority groups to specialty practices, unless they personally know a culturally competent clinician to whom they can refer. For example, a PCP pointed out,

I experience … overt barriers within the clinic for the patients, the cultural differences of that clinical climate and how the patients are spoken to and how they feel there. I really have to navigate this hospital to find providers that meet the patients where they are and how they feel.

An abundance of cardiologists with available appointments does not equate to access for racial and ethnic minority patients if the cardiologists are not culturally competent. As a vascular medicine specialist stated,

We don't really teach our physician/nurse practitioner/PA clinicians how to diagnose [PAD] in people who have different skin types, skin colors, race, [and] ethnicity.

Participants emphasized that patients, particularly those with no or poor insurance, may be forced to navigate a fragmented health care system with little help. This worsens disparities. Lack of transportation and other socially related challenges were noted as reducing access to necessary services, especially when multiple appointments are required at different locations. Participants pointed out that public awareness and education are lacking around lower-extremity PAD and its consequences, which may disproportionately impact people with low health literacy. A nurse practitioner commented,

… [public] awareness [for PAD] needs to go up, I've seen commercials for neuropathy and medications to treat neuropathy. I've never seen a commercial for PAD … more awareness [is needed] as a … public service.

### Solutions

Despite the challenges discussed in the roundtables and interviews, several case examples of solutions emerged that may serve as a guide for health systems trying to improve early detection and management of PAD.

#### Case Study 1: Value-based care approach: Benefit to the health system and to each patient

In a care system that has aggressively pursued risk contracts in an urban environment, it was a financial analysis of a proposed limb salvage program that highlighted the need to address PAD further upstream, focusing on earlier diagnosis and secondary prevention. This organization is a not-for-profit integrated health system that spans 2 states in the Midwest. It is one of the largest network providers of cardiovascular services in the region and has a top-decile heart and vascular program. Their director of managed care reported,

… when we really looked at our population health data, we saw that the biggest need was [in] our vulnerable communities where [there are] significant barriers [related] to social determinants of health and access to care.

They increased focus on early detection of lower-extremity PAD by identifying tools and resources for use in primary care, including detailed protocols that covered the physical examination and diagnostic criteria. They developed internal guidelines for screening, as well as treatment aimed at preventing advanced disease. For example, primary care practices received “in-network” resource lists for structured exercise programs to which they could refer patients, removing the burden of searching for an appropriate covered program from the PCP and the patient.

Once established in their main campus primary care clinics, the health system rolled out education and resources to primary care clinics located in under-resourced communities, encouraging them to actively identify and evaluate asymptomatic patients with risk factors. They also incorporated bedside volume plethysmography developed for PAD screening similar to a pulse oximeter (QuantaFlo^®^; Semler Scientific). This device compares blood flow in the toes to that in the fingers and requires much less time, training, and equipment than an ABI, and displays waveforms that help validate placement of the sensor.

Two studies demonstrated that a positive screening result for previously undetected lower-extremity PAD was associated with risk for major adverse limb events and major adverse cardiovascular events suggesting volume plethysmography may be used to risk-stratify patients for further evaluation.^25,26^ Thinking from a population health perspective, they realized they could screen asymptomatic patients at high risk without reimbursement because the long-term return on investment from preventing or delaying advanced disease would be both clinically and economically favorable.

In addition to lower future health care utilization among patients identified with early PAD, another component of the financial model was a focus on accurate Hierarchical Condition Category (HCC) coding. Taking on financial risk for patients requires their conditions be completely and accurately coded, to receive appropriate payment rates, which in turn cover the cost of necessary assessments and preventive care such as supervised exercise and smoking cessation counseling.

#### Case Study 2: Finding an advanced practice provider champion

One organization placed within their vascular surgery service an advanced practice provider (APP), a nurse practitioner, who is focused on vascular medicine. This organization is the largest multispecialty medical group in Mississippi, with 450 physicians and APPs. The group has >50 medical departments and specialty services and has >60 satellite locations, enabling them to offer care to individuals who live in rural areas and small towns.

Beyond the traditional narrow roles of assessing the need for surgical intervention and pre- and postprocedure management, this APP explicitly focuses on the evaluation and medical management of early PAD, supported with mentoring and clinical guidance from internal medicine. She has emerged as a champion for PAD within the group. Her location within vascular surgery facilitates additional testing when needed for further evaluation, avoiding unnecessary delays and travel burdens for patients.

Several more APPs have been added, to accommodate a growing number of referrals to vascular surgery for evaluation and to manage patients discovered to have early PAD. They spend time with patients conducting tasks for which the PCP may not have time—a careful history, detailed physical examination, and working with the vascular technicians who perform ABIs. The APP uses motivational interviewing around supervised exercise and smoking cessation, intensifies statins, and prescribes dual-pathway pharmacotherapy as appropriate.

Medical intervention is thought to be resulting in fewer repeat interventions, but that has not been rigorously studied. The population focus on PAD has increased referrals for evaluation from PCPs and specialists alike. Evaluation after referral for 1 vascular bed often leads to the discovery of atherosclerotic disease elsewhere, requiring further evaluation and management.

A key element of this model is a “travel clinic” that outreaches to rural clinics across the region, including primary care, wound care, and podiatry. This provides the opportunity to see more patients but more importantly to connect with clinicians and provide education on signs and symptoms to detect lower-extremity PAD early, and when to refer to vascular surgery. There is a recognized need for education of both patients and clinicians, who may not be receiving sufficient PAD-specific education during their training.

The APP has adapted education to be patient-centered and appropriate for low health literacy, relying on pictures and diagrams for simple explanations of the disease process. Finally, the APP is trained and has the support to coordinate care for the patient, for example, appointment scheduling including telehealth, and addressing transportation issues, which include limitations of Medicaid-funded transport services with advance reservation requirements, hindering the ability to get patients in quickly for needed testing. This attention to practical issues of coordination may reduce disparities in care. As 1 cardiologist/vascular medicine specialist stated,

Having a pathway for someone to receive care after they are diagnosed is key … from a population health standpoint you're identifying early disease, preventing late complications, and improving overall health … I think there's a lot of opportunity for wins if you can get somebody to step up and say I'm the champion.

#### Case Study 3: Leveraging multispecialty multidisciplinary teams

Integration of care into a multispecialty multidisciplinary clinical environment was cited by the vascular medicine service of an academic medical center as solution to improve the diagnosis, treatment, and management of lower-extremity PAD and to help reduce disparities. Multispecialty clinics are easier for patients to navigate, with all specialists available in a single setting. A patient with PAD can see the vascular surgeon, endocrinologist, podiatrist, and wound care nurse, all in a single location.

Transportation and taking time off work are less of an issue for patients when appointments can be coordinated across specialists or when telehealth options are available. This group has found that periodic check-ins and motivational interviewing work well through telehealth. They rely on clinical pharmacists for holistic interaction with patients who have PAD, a role that is broader than medication management. They use a patient registry to support care coordination and outreach, enabling the care team to close gaps in care by systematically tracking patient interactions in a format that is accessible to all providers and staff.

Efforts to make the overall health system work better provide greater benefit to those whom the current system serves least well—racial and ethnic minority groups and other socioeconomically disadvantaged patients. Colocation benefits clinicians as well, who can more easily discuss cases and coordinate care. For all these reasons, this site reported very good rates of follow-up in their multidisciplinary clinic.

## Discussion

Professional organizations are prioritizing lower-extremity PAD and the disparities it reveals. In November 2020, an AHA Presidential Advisory declared that structural racism causes poor health and premature death from heart disease and stroke.^27^ In March 2021, AHA announced a policy goal of reducing nontraumatic lower-extremity amputations by 20% by 2030.^28^

There are now several reasons for health systems to prioritize early evaluation and diagnosis of lower-extremity PAD, with recent evidence for dual-pathway medical therapy and recent expansion of payment for structured exercise and smoking cessation.

A key obstacle highlighted by participants in this research is the difficulty of identification of PAD in patients due in part to the impracticality of performing ABIs, the initial standard test for diagnosing PAD. Other studies have reported similar constraints such as lack of time, reimbursement, staff availability, equipment availability, and training as barriers to PAD screening and ABI measurement.^29,30^ Participants in this study provided many practical examples to overcome these obstacles, which included embedding an APP champion into practices to evaluate and medically manage patients with PAD and to educate other health care providers; use of alternative diagnostic devices; and multidisciplinary teams and clinics.

From a population health perspective, a more accurate and complete diagnostic picture provides a clearer sense of overall cardiovascular risk. In patients without other serious cardiovascular diagnoses, diagnosis of PAD can affect payments that depend on HCC coding, potentially making more resources available to support care coordination and to address health equity.

The rationale for why health systems and providers should invest in prevention and early detection of PAD relies on value-based payment, which involves a provider organization assuming financial risk for a patient population and thereby paying close attention to unnecessary or avoidable health care utilization. The limb salvage program described in Case Study 1 provided 1 example. However, a lack of risk-sharing options available to providers may limit the adoption of these solutions.

Medicare Advantage now accounts for 51% of Medicare beneficiaries^31^ and provides a fully capitated payment to the health plan, but many health plans still contract with providers on a fee-for-service basis. Fewer than one third of patients enrolled in traditional Medicare are assigned to providers participating in the Medicare Shared Savings Program (MSSP).^32^ This leaves >40% of adults >65 years in fee-for-service programs, giving provider organizations little financial incentive to prioritize preventive care.

Commercial insurers tend to lag behind Medicare and Medicaid in offering to share financial risk with providers, and there are significant regional differences in insurers offering risk-based contracts to providers.^33^ This may explain the historical and even the current lack of focus on early PAD, but value-based payment is steadily expanding, aligning financial incentives with improving population health. Early identification of PAD presents a substantial opportunity for achieving cost savings through secondary prevention, with timely medical therapy.

Moreover, as organizations consider ways to advance health equity, PAD provides an ideal opportunity—a common condition that is currently characterized by serious disparities in care and in patient outcomes. As confirmed by study participants, improving the diagnosis and management of PAD could have a substantial impact in reducing disparities.

### Limitations

There are limitations to qualitative research that apply to this study. The authors believe their roundtables and interviews approached an asymptote in identifying new issues, but the sample size was limited and may not be representative of all providers in the United States. The researchers' presence during the roundtables and interviews, although unavoidable, may have affected subjects' responses, although efforts were made to elicit candid responses. Finally, as much as we “member-checked” the data, there still remains the possibility that a particular participant's response was misinterpreted.

## Conclusion

Although several challenges to early detection and treatment of lower-extremity PAD were identified, including a lack of population health prioritization, little primary care focus, lack of ownership of the early detection of disease, and disparities in care, several solutions were offered. Designating and embedding a vascular medicine APP champion in a vascular surgery service, making the argument for a population health focus in value-based care environments, and leveraging multispecialty multidisciplinary teams, were suggested as viable solutions.

Multidisciplinary as well as travel clinics were reported to reduce disparities in care for PAD patients including those for whom transportation and time off work were major barriers to accessing care. Education of both patients and providers and other clinic staff is sorely needed. Each of these solutions contribute to the reduction in disparities in the early diagnosis and treatment of PAD.
